# Closing the genome of *Teredinibacter turnerae* T7902 by long-read nanopore sequencing

**DOI:** 10.1128/mra.00484-24

**Published:** 2024-12-10

**Authors:** Mark T. Gasser, Annie Liu, Ron Flatau, Marvin A. Altamia, Claire Marie Filone, Daniel L. Distel

**Affiliations:** 1 Johns Hopkins University Applied Physics Laboratory, Laurel, Maryland, USA; 2 Ocean Genome Legacy Center, Northeastern University, Nahant, Massachusetts, USA; Indiana University, Bloomington, Indiana, USA

**Keywords:** shipworm, symbiosis, endosymbionts, intracellular bacteria

## Abstract

We present the complete closed circular genome sequence derived from the Oxford Nanopore sequencing of the shipworm endosymbiont, *Teredinibacter turnerae* T7902 (DSM 15152, ATCC 39867), originally isolated from the shipworm, *Lyrodus pedicellatus* (1). This sequence will aid in the comparative genomics of shipworm endosymbionts and the understanding of the host–symbiont evolution.

## ANNOUNCEMENT

*Teredinibacter turnerae* is a cellulolytic gammaproteobacterium (Cellvibrionaceae) that occurs as an intracellular endosymbiont of wood-boring bivalves (Teredinidae) (1–4) commonly known as shipworms. Strain T7902, representing *T. turnerae* clade II (5, 6), was isolated from the gills of the shipworm, *Lyrodus pedicellatus*, collected in Long Beach, CA in 1979 (1). Briefly, the gills were homogenized in shipworm basal medium (SBM) (7) and streaked on 0.9% agar SBM plates supplemented with 0.2% w/v powdered cellulose (Sigmacell Type 100; Sigma-Aldrich) and 5 mM NH_4_Cl. Colonies were then serially re-streaked to obtain a clonal isolate. The original genome sequence of *T. turnerae* T7902 was published to GenBank (GCA_000379165.1) but was not described in peer-reviewed literature. This sequence was completed on 2012–05-22 at the DOE Joint Genome Institute under award 10.46936/10.25585/60001419 using 454 GS FLX Titanium and Illumina HiSeq 2000 sequencing platforms. It was assembled using Velvet v. 1.0.13 (8) and ALLPATHS v. R40295 (9), resulting in an improved high-quality draft assembly comprising 72 scaffolds with 76 contigs. As of 2024–05-10, eight fragmented genomes and one closed circular genome (T7901, Clade I, GenBank: GCA_000023025.1) were available for strains of *T. turnerae*. Here, we present the re-sequencing and completed genome of strain T7902 from nanopore-only sequencing (Table 1).

**TABLE 1:** Assemblies of *Teredinibacter turnerae* T7902

GenBank Assembly	Scaffolds (contigs)	Size (bp)	GC%	CDS
GCA_000379165.1	72 (76)	5,387,817	50.8	4268
GCA_037935975.1 (this work)	1 (1)	5,348,823	50.9	4212

**Fig 1:**
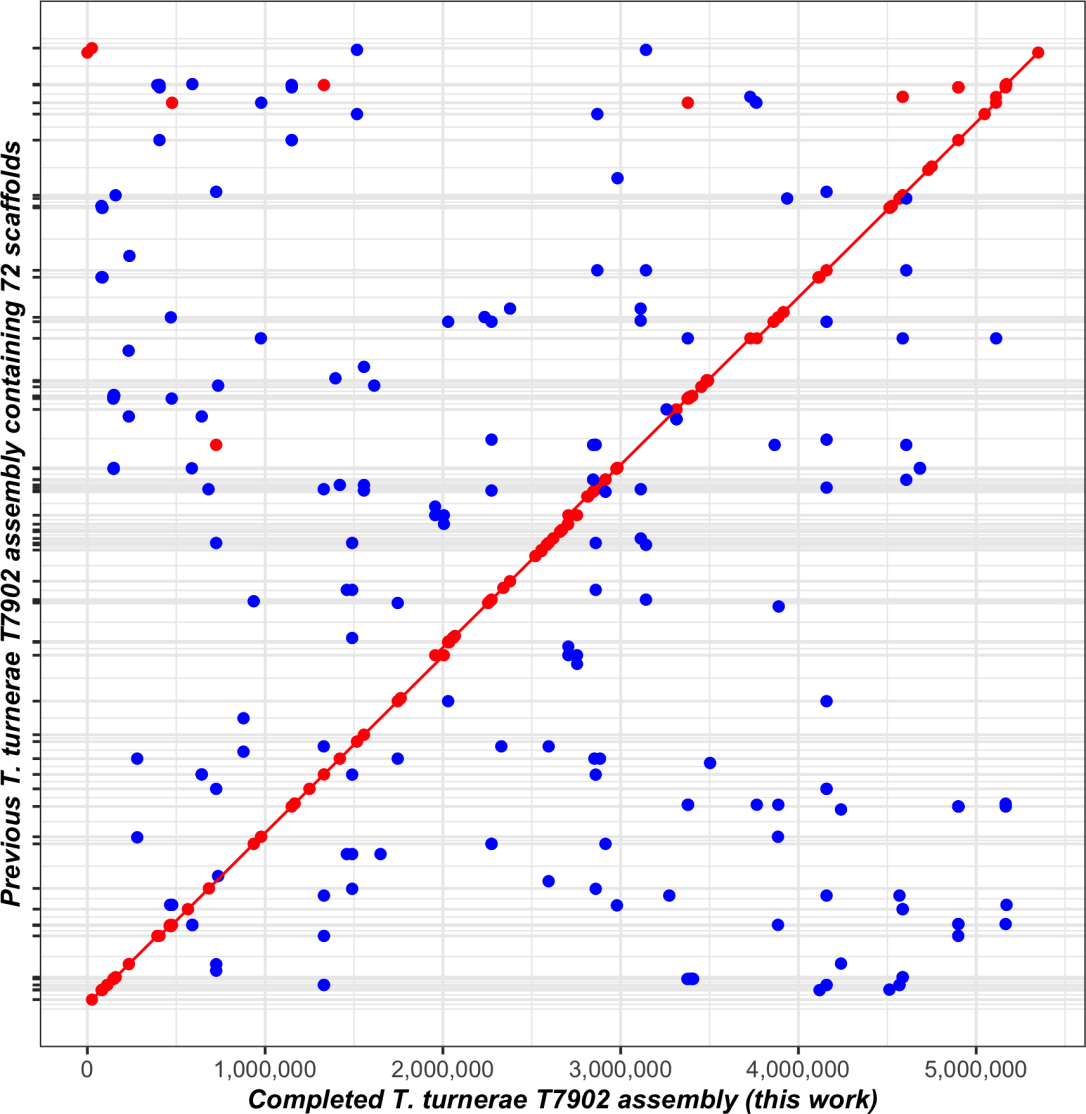
Synteny plot comparing the previously published genome of *Teredinibacter turnerae* T7902 (GCA_000379165.1) and the new genome sequence and assembly presented here (GCA_037935975.1). A MUMmer3 plot was generated with NUCmer v3.1 (15) using default settings to assess synteny and completion. Minimum exact matches of 20 bp are represented as a dot, with lines representing exact match lengths > 20 bp. Forward matches are displayed in red, while reverse matches are shown in blue.

A colony of *T. turnerae* strain T7902 grown at 30°C on SBM (7) plates supplemented with 0.025% NH_4_Cl and 0.2% cellulose (Sigmacell Type 101; Sigma-Aldrich) was used to inoculate a 6 mL liquid culture of SBM supplemented with 0.025% NH_4_Cl and 0.2% carboxymethyl cellulose medium and grown at 30°C, 100 rpm for 4 days. Bacterial cells were harvested by centrifugation (10 min, 4°C, 4,000×*g*), and high-molecular-weight DNA was isolated from the cell pellet using the Wizard HMW DNA Extraction Kit (Promega, US) according to the manufacturer’s protocol. The DNA quality and length were assessed on a Tapestation DNA Analyzer (Agilent Technologies, US). Nanopore sequencing (Oxford Nanopore Technologies, UK) was performed by ligation using the Nanopore Q20+ Chemistry Kit v14 according to the manufacturer’s protocol and sequenced on a MinION instrument using an R10.4 flow cell(FLO-MIN112). Bases were called using Guppy v6.5.7 with the super-accurate (SUP) algorithm and default-quality read filtering generating 300,309 reads (*N*_50_ = 8,763 bp). *De novo* assembly was performed with Flye v2.9.2 (https://github.com/fenderglass/Flye) (10), followed by contig correction and consensus generation with Medaka v1.8.0 (https://github.com/nanoporetech/medaka). To circularize, overlaps were identified and removed before the assembly was rotated to the gene predicted by Prodigal v2.6.3 (11) nearest the middle of the contig with Circlator v1.5.5 (https://github.com/sanger-pathogens/circlator) (12). The resulting chromosomal assembly (278.0× coverage) was annotated using the NCBI Prokaryotic Genome Annotation Pipeline (13). The new assembly shares 99.99% average nucleotide identity (14) and is highly syntenic (15) with the original (Fig. 1) but reduces the genome size by 38,994 to 5,348,823 bp, contains 56 fewer predicted CDS, and resolves several assembly errors. For all software, default parameters were used, except where otherwise noted.

## Data Availability

The complete genome sequence of T7902 has been deposited in GenBank under the accession number CP149817. The Oxford Nanopore sequencing reads are available from the NCBI Sequence Read Archive (SRA) under the accession number SRR28421272.
